# Cetuximab-Associated Crescentic Diffuse Proliferative Glomerulonephritis

**DOI:** 10.1155/2017/7964015

**Published:** 2017-11-16

**Authors:** Sukesh Manthri, Sindhura Bandaru, Anthony Chang, Tamer Hudali

**Affiliations:** ^1^Southern Illinois University, Springfield, IL, USA; ^2^The University of Chicago Medicine, Chicago, IL, USA

## Abstract

Cetuximab-induced nephrotoxicity is very rare, occurring in less than 1% of colorectal cancer patients and not defined in other populations. We report a rare case of crescentic diffuse proliferative glomerulonephritis (GN) that developed in close temporal association with cetuximab treatment. A 65-year-old female recently completed chemotherapy with cetuximab treatment for moderately differentiated oral squamous cell carcinoma. She was admitted with acute renal failure and nephrotic-range proteinuria. Laboratory data showed serum creatinine of 6.6 mg/dl and urinalysis showed proteinuria, moderate hemoglobinuria, hyaline casts (41/LPF), WBC (28/HPF), and RBC (81/HPF). Serologic studies were negative for ANA, anti-GBM, ANCA, hepatitis B, and hepatitis C. Serum C3 and C4 level were normal. Renal biopsy showed crescentic diffuse proliferative GN with focal features of thrombotic microangiopathy. Patient was started on cyclophosphamide and steroids. Her renal function did not improve on day 8 and she was started on hemodialysis. Previous reports suggest that EGFR-targeting medications can possibly trigger or exacerbate an IgA-mediated glomerular process leading to renal failure. This case suggests that cetuximab therapy may have triggered or exacerbated a severe glomerular injury with an unfavorable outcome. Treating physicians should maintain a high degree of caution and monitor renal function in patients on EGFR inhibitors.

## 1. Introduction

Cetuximab is a genetically engineered mouse/human chimeric immunoglobulin G1 (IgG1) monoclonal antibody, which specifically binds to epidermal growth factor receptor, and is used for locally advanced, recurrent, and/or metastatic squamous cell carcinoma of the head and neck [1] along with advanced colorectal cancer [2]. Cetuximab-induced nephrotoxicity is rare, occurring in less than 1% in colorectal cancer patients. We report a rare case of crescentic diffuse proliferative glomerulonephritis developed in close temporal association with cetuximab treatment for oral squamous cell cancer.

## 2. Case Description

A 65 -year-old Caucasian female with stage T4aN2b moderately differentiated squamous cell carcinoma of right retromolar trigone was admitted for acute kidney injury. The squamous cell carcinoma was discovered 12 weeks prior to admission and required radical neck dissection and postoperatively she received 7 cycles of cetuximab and 33 sessions of radiation treatment. Her last dose was three weeks prior to kidney injury and nephrotic syndrome (renal function during cetuximab therapy is shown in Table 1). Other pertinent medical history includes asthma and hypertension. Her home medications include Losartan and Ventolin HFA inhaler.

Upon admission, patient complained of nausea and loose stools, at least 4-5 bowel movements per day. She denied any abdominal pain, fever, chills, or vomiting. Due to acute illness, patient was poorly hydrated. She was afebrile and slightly hypertensive (blood pressure, 146/76 mmHg), and physical examination showed 1+ pedal edema but otherwise was unremarkable. Laboratory data on admission showed an increased serum creatinine level at 6.6 mg/dl (estimated GFR, 6 mL/min/1.73 m^2^) from baseline of 0.7 mg/dl (estimated GFR, 84 mL/min/1.73 m^2^). Complete blood count showed the following values: white blood cells, 11.8 × 10^3^/uL; hemoglobin, 6.0 g/dL (Hgb was around 10.4 g/dL during 7 cycles of chemotherapy); hematocrit, 18%; and platelets, 332 × 10^3^/uL. Serum iron, ferritin, TIBC, and % saturation levels were consistent with anemia of chronic disease. She received 2 units of packed RBC transfusion and later her Hgb was stable at 9 gm/dL. Urinalysis showed proteinuria (>500 mg/dl), moderate hemoglobinuria, hyaline casts (41/LPF), WBC (28/HPF), and RBC (81/HPF). FeNa was 2.6% and urine protein-creatinine ratio was 12.29 g/g (nephrotic-range proteinuria). Serum albumin level was 2.4 g/dL. Serologic studies were negative for antinuclear antibodies, anti-glomerular basement membrane antibodies, anti-neutrophil cytoplasmic antibodies, hepatitis B, and hepatitis C. Serum C3 and C4 levels were normal at 88 mg/dL (reference range, 79–152 mg/dL) and 28 mg/dL (reference range, 13–38 mg/dL), respectively. Serum and urine protein electrophoresis results were negative for monoclonal proteins. Serum-free light chain assay showed elevated free kappa light chains of 477 mg/L (reference range, 3.3–19.4 mg/L) and lambda light chains of 321 mg/L (reference range, 5.7–26.3 mg/L), but ratio was normal. Serum-free light chains can be 20–30-fold above the upper limit of normal in patients with acute kidney injury [3]. Bilateral renal ultrasound showed increased renal parenchymal echogenicity bilaterally, mild bilateral caliectasis, small amount of ascites, and bilateral pleural effusions. She underwent kidney biopsy to determine the cause of acute kidney injury.

**Figure 1:**
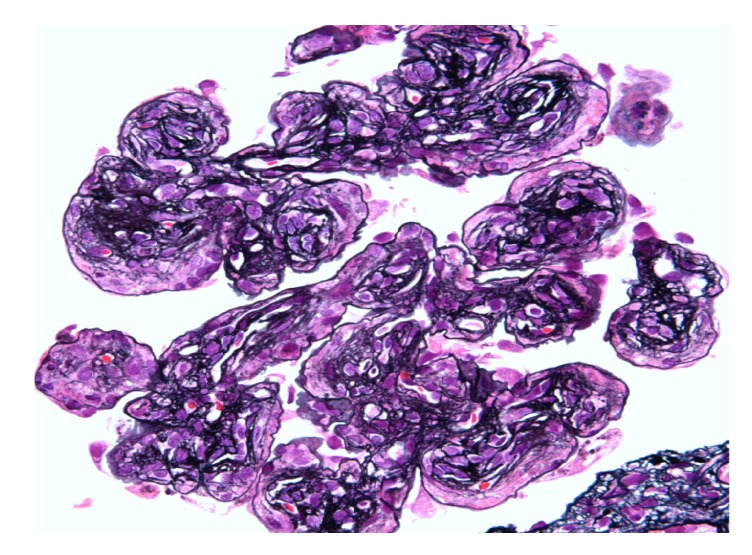
This glomerulus demonstrates accentuation of the lobular architecture with associated endocapillary hypercellularity and duplication of the glomerular basement membranes (Jones methenamine silver).

**Figure 2:**
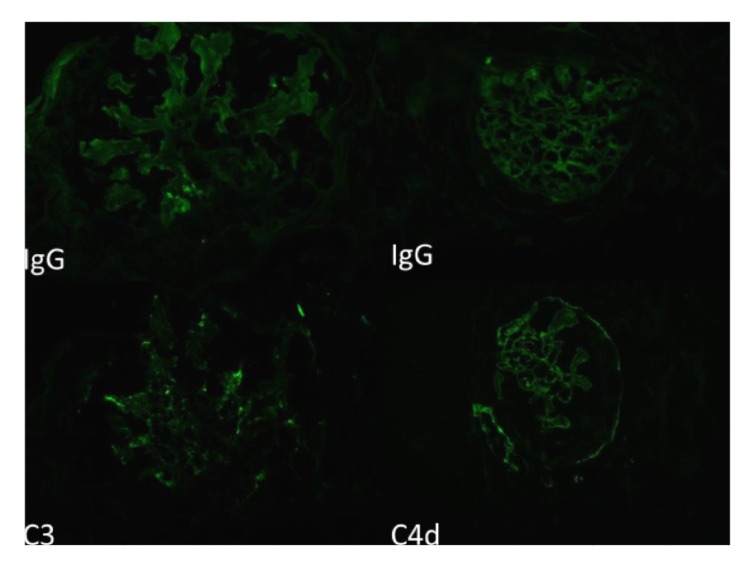
There is granular mesangial and capillary wall immunofluorescence staining for the respective immunoglobulins and complement components that range from 1 to 2+ on a scale of 0–4+.

**Figure 3:**
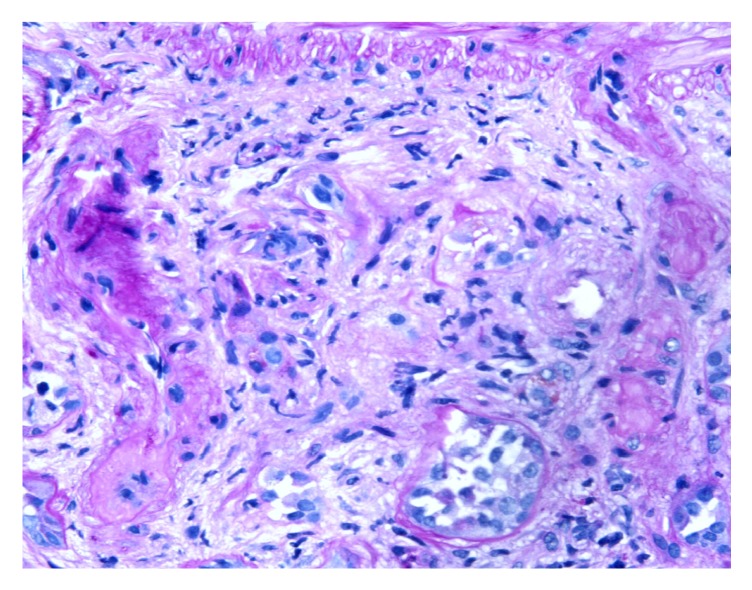
Figure demonstrating arteriolar thrombotic microangiopathy.

**Figure 4:**
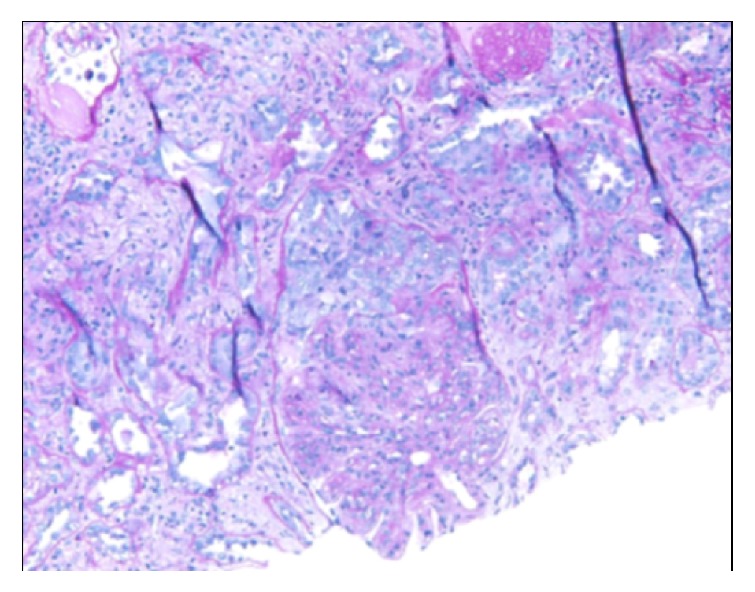
A cellular crescent fills Bowman space adjacent to this glomerulus with prominent endocapillary hypercellularity (Periodic acid-Schiff).

Kidney biopsy showed cellular crescents involving up to 63% of the sampled glomeruli (Figure 4), which demonstrated a membranoproliferative pattern of injury with prominent accentuation of the lobular structure and duplication of the glomerular basement membranes (Figure 1). Additional findings of a thrombotic microangiopathic injury were observed in a hilar arteriole with a thrombus (Figure 3). There was also diffuse interstitial edema and inflammation, consisting of lymphocytes, neutrophils, and some eosinophils, which were more prominent in the medulla. The degree of interstitial fibrosis and tubular atrophy was difficult to assess given the degree of tubulointerstitial inflammation. On a 0–4+ scale, immunofluorescence microscopy demonstrated granular capillary wall and mesangial staining for C3 (2+), C4d (1-2+), C1q (1+), and IgG (1+ in a segmental distribution) (Figure 2). A glomerulus with a crescent showed segmental fibrinogen (2-3+) staining. There was no significant glomerular staining for IgA, IgM, or kappa or lambda light chains. Glomeruli were not available in the sample submitted for electron microscopy and an attempt to process the paraffin tissue block for electron microscopy did not yield any additional glomeruli. The final diagnosis was crescentic diffuse proliferative glomerulonephritis with focal features of thrombotic microangiopathy.

The patient presented with a creatinine of 6.6 mg/dl which did not improve significantly with hydration, resulting in the initiation of hemodialysis after 7 days. Based on the biopsy findings, she was started on cyclophosphamide and pulse methylprednisolone followed by a taper and later discharged home. Unfortunately, she was readmitted to hospital 4 weeks later due to acute hypoxic respiratory failure secondary to MRSA pneumonia. She continued to be on scheduled hemodialysis and her immunosuppressive medications were temporarily held due to infection. Given her poor prognosis, the family chose comfort measures and the patient died.

## 3. Discussion

The number of targeted therapies for advanced cancers is rapidly increasing. While their side effect profiles have been established, the increasing use of these agents will reveal rare complications, such as those in our patient. Checkpoint inhibitors, immunomodulatory antibodies that are group of novel drugs that are used to enhance the immune system, are substantially being used. Cetuximab can frequently cause electrolyte imbalance (hypomagnesemia, hypokalemia, and hypocalcemia) and renal tubular acidosis. Our biopsy represents the third report of glomerular injury associated with cetuximab, given the close temporal association of therapy. The crescentic membranoproliferative pattern of injury with an additional component of a thrombotic microangiopathic injury represents the most severe complication of cetuximab that has been yet reported.

The protein construct for cetuximab consists of Immunoglobulin G1, anti-human epidermal growth factor receptor (human-mouse monoclonal C225 g1-chain), disulfide with human-mouse monoclonal C225 kappa-chain, dimer [4]. The IgG that was detected by immunofluorescence microscopy in the biopsy may represent the recombinant protein itself, even though monoclonal kappa light chain staining was not detected, as the modest degree of IgG staining could be related to the three-week interval since the last administration. This has been demonstrated to occur with eculizumab [5]. Anti-chimeric (or human anti-mouse) antibodies were found in just under 4% of patients being treated with cetuximab in phase 1 trials [6]. It is possible that this patient was one of the rare patients to develop these antibodies and that these were responsible for her renal failure. Unfortunately, we could not test for these antibodies.

Glomerular diseases are associated with many solid and hematologic malignancies. The exact pathogenesis of these glomerular lesions is unclear. Membranous nephropathy and IgA nephropathy are reported glomerular diseases associated with head and neck cancer. Crescentic glomerulonephritis has been associated with renal cell, gastric, and lung cancers [7]. Cancer-associated glomerular diseases have shown that treating the cancer may lead to resolution of the glomerular process. Our patient developed GN after finishing treatment with targeted therapy. She responded very well to cancer treatment and she was in remission. So we suspect her glomerular injury to be in close temporal association with cetuximab treatment.

We are aware of two reports of glomerular injury associated with cetuximab. Sasaki et al. [8] described IgA-dominant proliferative and crescentic glomerulonephritis in a patient treated with cetuximab. In that patient kidney function dramatically improved with cessation of cetuximab and a short course of cyclophosphamide and methylprednisolone. Unlike this case, our patient's renal function did not improve after 4 weeks of immunosuppressive therapy. Koizumi et al. [9] reported a kidney biopsy with glomerular features of TMA due to cetuximab administration. Administration of cetuximab was discontinued and, nine weeks after the discontinuation of cetuximab, the proteinuria decreased.

Our case is unique with both crescentic diffuse proliferative glomerulonephritis associated with focal features of thrombotic microangiopathy (TMA). TMA is usually common to hemolytic uremic syndrome, atypical hemolytic uremic syndrome, and thrombotic thrombocytopenic purpura and can also be induced by various drugs. Radiation treatment could contribute to this injury process, but our patient's radiation was localized to the head and neck region without direct exposure of the kidneys. The possibility of direct endothelial cell toxicity by cetuximab cannot be entirely excluded. Atypical hemolytic uremic syndrome is difficult to exclude, but there was no overt evidence of microangiopathic hemolytic anemia or thrombocytopenia.

Steroids combined with cyclophosphamide is the mainstay of treatment for MPGN reported in case reports. Our patient's renal function did not improve on day 8 of hospitalization and she was started on hemodialysis along with a short course of methylprednisolone and cyclophosphamide.

Although glomerular injury in patients treated with EGFR inhibitors is rare (<0.01%) and no cases were reported in randomized clinical trials using EGFR inhibitors, [10] this may be an underappreciated complication, as kidney biopsies are not often obtained, so increased awareness and close monitoring of kidney function in these patients is warranted.

## Figures and Tables

**Table 1:** Patient's sodium (mmol/l), potassium (mmol/l), BUN (mg/dl), creatinine (mg/dl), serum albumin (gm/dl), proteinuria (mg/dl), and hematuria (per hpf) during 7 cycles of cetuximab treatment and 3 weeks later after completing 7th cycle of treatment.

	1st cycle	2nd cycle	3rd cycle	4th cycle	5th cycle	6th cycle	7th cycle	3 weeks later
Sodium	130	133	133	136	133	130	131	134
Potassium	4.5	4.9	4.4	4.6	4.4	4.5	4.3	4.1
BUN	20	24	25	30	19	19	18	*100*
Creatinine	0.8	0.7	0.7	0.7	0.7	0.8	0.8	*6.6*
Serum albumin	3.2	3.7	3.5	3.3	3.0	3.1	2.4	*1.5*
Proteinuria	30	n/a	n/a	n/a	n/a	n/a	n/a	*>500*
Hematuria	1	n/a	n/a	n/a	n/a	n/a	n/a	*81*
